# Racial differences in the associations between adiposity, placental growth hormone and inflammatory cytokines in pregnant women

**DOI:** 10.3389/fendo.2023.1100724

**Published:** 2023-03-17

**Authors:** Camille Y. Williams, Amanda Wylie, Verina Ghobrial, Christopher L. Coe, Sarah J. Short

**Affiliations:** ^1^ Department of Counseling Psychology, University of Wisconsin–Madison, Madison, WI, United States; ^2^ Center for Healthy Minds, University of Wisconsin–Madison, Madison, WI, United States; ^3^ Frank Porter Graham Child Development Institute, University of North Carolina at Chapel Hill, Chapel Hill, NC, United States; ^4^ Department of Psychology and Neuroscience, University of North Carolina at Chapel Hill, Chapel Hill, NC, United States; ^5^ Department of Educational Psychology, University of Wisconsin–Madison, Madison, WI, United States; ^6^ Harlow Center for Biological Psychology, University of Wisconsin–Madison, Madison, WI, United States

**Keywords:** placental growth hormone, obesity, inflammation, pregnancy, Black or African American, birthweight

## Abstract

**Background:**

The prevalence of obesity among women of child-bearing age has contributed to an increased risk of pregnancy complications with a disproportional impact on women of lower socioeconomic status and among certain racial groups. In particular, socio-demographic and historical factors have resulted in higher rates of premature births and small-for-gestational age infants among Black women, which may be associated with placental function during pregnancy. The current study investigated the influence of maternal pre-pregnancy adiposity and race on the associations between inflammatory proteins, placental growth hormone (PGH), and infant birthweight. This information was collected for a subsample of 109 participants (Black, n = 39 vs. White, n = 70) from the Brain and Early Experiences (BEE) study.

**Methods:**

Serum samples were acquired late in the second trimester to assess PGH levels, C-reactive protein (CRP), interleukin 6 (IL-6), interleukin 8 (IL-8), and interleukin-1 receptor antagonist (IL-1Ra). Participant questionnaire responses provided information on pre-pregnancy BMI, health, race, educational attainment, and infant birthweight. Bivariate correlations and multiple linear regression models were utilized to evaluate associations by race between preconception adiposity, inflammatory markers and PGH.

**Results:**

After controlling for covariates including maternal age and education, gestational age, and fetal sex, regression models indicated that pre-pregnancy BMI was negatively associated with PGH (*β*=-0.42, *p*<0.05) and IL-8 was positively associated with PGH (*β*=0.35, *p*<0.05) among the Black mothers only; neither were significantly associated with PGH in the White mothers. When extending models to birth outcomes, BMI was positively associated with birthweight corrected for gestational age (BWz) (*β*=0.24, *p*<0.05) and educational attainment was negatively associated with BWz (*β*=0.28, *p*<0.05) for infants of White women. In contrast, neither variable was predictive of BWz for infants of Black mothers.

**Conclusion:**

Future work is needed to investigate racial differences in the association between adiposity and placental functioning, which are likely to contribute to differential effects on pregnancy outcomes and fetal growth.

## Introduction

Racial and ethnic differences in the prevalence of pregnancy and birth complications reflect historical, social, and economic disparities, and there is evidence that these disparities continue to contribute to transgenerational health inequities. In particular, Black women experience more pregnancy complications and have premature births and small-for-gestational age infants at a disproportionately high rate—nearly twice that of White women (1). These differences are thought to be due to inequalities in education, income, housing, access to healthcare, and the stress caused by overt racial discrimination (2). In addition, the higher prevalence of obesity among Black women of reproductive age appears to be a significant risk factor for gestational hypertension and diabetes. There is increasing evidence that obesity during pregnancy and the pathophysiology of hypertension and gestational diabetes can affect both the structure and function of the placenta, with adverse consequences for the developing fetus (3). This concern was investigated in the present analyses, which focused on a key hormone synthesized and released by the placenta, placental growth hormone (PGH). Further, both basic science studies in animals and clinical research in humans indicate that obesity can accentuate inflammatory physiology during pregnancy, which also impacts placental functions important for fetal development. Several bioindicators of inflammatory activity were also assessed in this study, including C-reactive protein and the levels of proinflammatory cytokines in maternal circulation.

During pregnancy, the placenta plays a key role secreting hormones and immune-related proteins essential for the regulation and maintenance of the gravid state until term. One of these important regulatory hormones is PGH, which was first discovered in the late 1980s (4, 5), and found to have significant effects on maternal metabolism. It is synthesized initially by the trophoblast cells that will become the placenta and then PGH levels increase across pregnancy, supplanting the normal pituitary secretion of maternal growth hormone (GH) during the second and third trimesters (4, 6, 7). Although less PGH is released directly into fetal circulation, it is detectable in amniotic fluid (4), and has several indirect but important effects on fetal growth. While its primary actions are on maternal physiology, modulating lipid metabolism and glucoregulation, it also affects angiogenesis and the fetal blood supply and enhances the availability of nutrient resources to the fetus (8). PGH has been used extensively in clinical research as a bioindicator of risk for hypertension and premature birth (9, 10). Typically, lower levels of PGH are found to be predictive of an unhealthy pregnancy and placental dysfunction, and have been associated with preeclampsia, gestational diabetes, and premature birth (11). However, its sensitivity as a biomarker that might also be associated with sociodemographic status and maternal adiposity has not been systematically evaluated. Similarly, the extent of the association between PGH and inflammatory activity during pregnancy – specifically between PGH and cytokines known to be released by the placenta, such as IL-8 – has not been established.

Maternal immune responses, including the release of cytokines, play an important role in the initiation and maintenance of pregnancy. They help to mediate the uterine engulfment of the implanting embryo and the sculpting of the blood vessels that will become the placental vasculature (12). Across pregnancy, the levels of many cytokines rise progressively in maternal circulation, and some cytokines, including both IL-6 and IL-8 have been found to be significantly higher in the third trimester (13). Although these cytokines have beneficial and supportive functions at normal levels, their actions may become detrimental when elevated, such as following bacterial infections in the case of chorioamnionitis, or after viral infections including SARS-CoV-2 (14–16). Proinflammatory cytokines, including IL-6, are associated with maternal obesity, because the IL-6 in maternal circulation is also synthesized and released by maternal adipocytes, in addition to being secreted by hepatocytes and placental tissue. IL-6 is a potent stimulator of CRP from the liver, and the levels of CRP in circulation also tend to be higher in pregnant women who are overweight. IL-8 in maternal circulation is known to be directly released from the placenta and IL-8 levels increase across pregnancy (17). Because IL-8 is high at term and during delivery, it has been hypothesized to help initiate labor. In addition, IL-8 is elevated in pregnant women with hypertension or preeclampsia and it is one of the chemotactic signals responsive to bacterial infection of placental tissue, including chorioamnionitis (18–22). Other studies have found that there may be a predictive relationship between higher levels of IL-8 during pregnancy and a risk for later psychiatric conditions in offspring (23). The present analyses examined the potential influence of pre-pregnancy adiposity and maternal race on the association between PGH and several soluble proteins that reflect inflammatory activity.

In addition to IL-6 and IL-8, IL-1 is another inflammatory cytokine that is relevant to understanding the relationship between adiposity and maternal prenatal health. However, because IL-1 levels are present at low levels in circulation, it is often more informative to quantify the levels of the soluble receptor antagonist for IL-1 (IL-1Ra) as a proxy for recent IL-1 release (24, 25). In addition, it has been found that the placenta will release IL-1Ra in a compensatory and regulatory manner to an increase in IL-1 activity (26, 27). Thus, our analyses focused primarily on IL-1Ra, IL-6, IL-8, and C-reactive protein (CRP), as indicators of maternal and fetal wellbeing (28–31). Although the importance of immunomodulatory proteins during pregnancy has been extensively documented in animal models (12), the relations between sociodemographic variables, PGH, and cytokines have yet to be examined systematically in pregnant women.

As highlighted above, pre-pregnancy adiposity is a known risk factor for pregnancy complications (32–34), and obesity is associated with proinflammatory activity in both nonpregnant and pregnant individuals (35). Children born to obese mothers are also at increased risk of obesity, metabolic disease, and neuropsychiatric disorders (36). Differences in the prevalence of obesity may contribute to some of the significant racial disparities in pregnancy outcomes (37, 38). Further, the risk of gestational hypertension and preterm birth in Black women has been linked to a dysregulation of inflammatory processes (39–41). However, a potential association with soluble proinflammatory factors in maternal circulation associated with placental function has not been clearly delineated. The specific aims of this study were to: 1) evaluate associations between maternal adiposity, inflammatory cytokines, and PGH late in the second trimester, 2) examine whether associations between maternal adiposity, inflammatory markers, and PGH differ by maternal race, and 3) assess whether these biological measures were indicative of infant weight at term. The *a priori* hypotheses were that a) maternal adiposity would be negatively associated with PGH levels, b) higher levels of one or more of the measured inflammatory proteins would be associated with lower levels of PGH, and c) lower PGH levels would be associated with lower infant birth weight. Race-related differences in the association between maternal adiposity and PGH levels, and PGH and inflammatory proteins, were unknown, and were thus examined as an exploratory aspect of this investigation.

## Methods

To address these goals, we leveraged data from the ongoing Brain and Early Experiences (BEE) Study—a longitudinal study of mother-infant dyads and their families in central North Carolina.

### Participants

Pregnant women were recruited through social media advertisements, flyers at local obstetric and gynecologist clinics, and the local Women, Infant, and Children offices to join the BEE Study. Pregnant women were eligible to join the study if they were 18 years or older, spoke English, had a singleton pregnancy, lived within 60 miles of the study site (Chapel Hill), and had no plans to move from the geographic area for the next three years. Pregnant women provided consent during their first study visit, at mid-late pregnancy when maternal measures were collected for this study. Women and their infants were officially enrolled in the lager BEE Study if the infant was born at 36 weeks and 4 days of gestational age (GA) or older, had a birth weight of at least 2.49 kg (5.5 pounds) without significant medical complications, had no metal devices implanted, and participated in at least two of the first three data collection visits (prenatal, 2-week postpartum, and 6-months postpartum).

### Procedures

This analysis focuses on a subsample (N=109) of mother-infant dyads who completed the prenatal laboratory visit prior to the COVID-19 pandemic and identified their race as Black or African American (referred to as “Black”) or White. While in mid-pregnancy (mean GA=27.1 weeks; SD=2.1 weeks), pregnant women came to the laboratory for a two-hour visit. Participants completed a series of questionnaires about themselves, their pregnancy, health, and relationships; a non-fasting blood sample was also obtained. A phlebotomist collected blood samples (12 mL) *via* venipuncture into two 6 mL EDTA tubes that were stored on ice. The samples were centrifuged for 10 min at 2000 x g at 4°C. Serum was aliquoted into 1 mL vials and stored at -80°C until time of assay. Participants were compensated $50 at the end of the prenatal laboratory visit. Mothers reported on birth characteristics, including infant birthweight and length at the 2-week postpartum study visit. The University of North Carolina at Chapel Hill Institutional Review Board (#17-1914) approved all study procedures.

### Measures

#### Prenatal Inflammation

CRP, IL-6, IL-8 and IL-1Ra were determined using an electrochemiluminescence platform and quantified with the MESO QuickPlex SQ120 instrument for analyte detection (Meso Scale Discovery, Gaithersburg, MD). Because CRP is present at high concentrations in circulation, it was determined in a singleplex assay. CRP was quantified in pg/mL units but reported as mg/L in keeping with clinical practice. The intra-assay CV for CRP was 2.8%. IL-1Ra was also assessed in a singleplex assay because this soluble receptor antagonist is present in the high pg/mL range, 500-1000 times the typical level of its ligand, IL-1. The intra-assay CV for the duplicate determinations averaged 3.5%. Two proinflammatory cytokines, IL-6 and IL-8, were selected for analysis from a 6-plex array that also included interferon-gamma (IFN-γ), interleukin-2, interleukin-10 and tumor necrosis factor-alpha. Each specimen was run in duplicate determinations and referenced to a standard curve generated from 7 calibrators with known cytokine concentrations. The lower limit of detection was: 0.06- 0.1 pg/mL with a wide dynamic range up to the lower ng/mL range. The intra-assay CV for IL-6 was 4.93%, and for IL-8 was 5.16%. Any value with a CV over 10% was re-run in a follow-up assay to verify the quantification. For the statistical testing, IL-6 was analyzed in fg/mL units so all values could be shown as positive integers after the log transformation. All inflammatory markers were natural log-transformed prior to analyses. Women who self-identified as Black had significantly higher IL-6 (*p* = 0.003) and lower IL-1Ra (*p* = 0.02) than women who self-reported as White; there were no significant differences in IL-8 or CRP by maternal race.

#### Placental growth hormone

Placental growth hormone (PGH) was quantified by high sensitivity enzyme-linked immunosorbent assay (RnD Quantikine ELISA) with a lower sensitivity of 3 pg/mL and upper limit of 1000 pg/mL. Samples (100 µL) were diluted 1:7 to ensure higher PGH values were quantified on the linear portion of the reference curve. Based on the duplicate determinations, the intra-assay coefficient of variation was 3.82%. PGH was natural log- transformed prior to analyses. There wasn’t a significant difference in PGH between pregnant women who identified as Black and those who identified as White.

#### Maternal pre-pregnancy BMI and weight gain

Pregnant women reported their pre-pregnancy weight and their height as part of a self-reported questionnaire completed during the prenatal laboratory visit. Maternal pre-pregnancy BMI was calculated as the participant’s weight in kilograms divided by their height in meters squared. Of the 108 women who reported their pre-pregnancy weight and height, the average BMI was 27.4 (SD = 6.7). Women who self-identified as Black had significantly higher BMIs than women who self-reported as White (p = 0.002).

#### Birthweight Z-Score

Infant birth weight, gestational age, and sex were acquired from the medical record to calculate a birthweight z-score (BWz) using the Fenton 2013 Preterm Growth Chart (42). If the medical record could not be accessed, we relied on maternal report of birth variables to calculate the BWz. A subset of participants declined further participation after the prenatal laboratory visit. As a result, for this analysis there were 99 birthweight z-scores, which averaged 0.0 (SD = 0.9). Although birthweight was significantly lower for the infants of Black mothers than White mothers (*p* = .049), after correction for gestational age at birth and the weight differences between female and male babies, the effect of race no longer attained statistical significance (*p* = 0.11).

#### Maternal race

As recommended by Martinez et al., race was considered on the basis of personally identified race (43). To capture the mother’s race, the women were asked “How would you describe your primary race?” with the options “Black or African American,” “White,” “American Indian, Alaskan Native, or Native Hawaiian,” “Asian Indian,” “Chinese,” “Japanese,” “Korean,” “Vietnamese,” “Other Asian,” or “Other” with an open-ended option. A two-category variable was generated, reflecting if the mother reported her primary race to be Black or African American (“Black”) or White.

#### Covariates

Educational attainment, maternal age at the prenatal visit, and gestational age at the prenatal visit were considered as covariates in the analyses. Because PGH levels have been reported to be higher in pregnancies with female fetuses (44, 45), we additionally adjusted for fetal sex in models examining only prenatal measures. These measures were acquired through maternal self-report as part of the prenatal visit and during birth checks (i.e., for maternal age at delivery). Adjustment for maternal education as an indicator of socioeconomic status was an important covariate, as previous studies have indicated that economic disadvantage may affect child health via its influence on inflammatory processes (46).

### Analytic strategy

Descriptive statistics for variables of interest, bivariate correlations between focal variables, and differences in continuous predictors by self-reported binary race were examined. Continuous variables were then standardized at mean of 0 and standard deviation of 1 facilitate the interpretation of regression coefficients.

To evaluate associations between preconception adiposity, inflammatory markers, and PGH (Aim 1), bivariate correlations and multiple linear regression models of PGH were examined. The models included gestational age at the prenatal visit, maternal age, maternal education, binary race, maternal pre-pregnancy BMI, IL-1Ra, IL-8, IL-6, CRP, and female sex. If an inflammatory marker was not statistically associated with PGH in the regression analysis, it was removed iteratively. We were conservative in selecting inflammatory markers, allowing markers that were statistically associated at an alpha = 0.1 to remain in the model.

To evaluate whether associations between adiposity, inflammatory markers, and PGH differed with respect to race (Aim 2), we stratified the sample by race as Black or White. This model was built with all inflammatory markers tested in Aim 1 and used the same variable reduction technique employed to build the final Aim 1 regression model.

To examine associations between PGH, adiposity, and inflammatory markers on the culmination of fetal growth (Aim 3), the final models from Aims 1 and 2 were extended to model BWz. In these regression models, PGH was included as an additional predictor variable but with gestational age at the prenatal visit and fetal sex removed from the model as fetal sex (as these are captured in the BWz variable). To evaluate whether associations differed with respect to sex, we stratified the sample by race. We also evaluated whether there might be mediating effects of BMI on BWz through PGH using the Process macro from Hayes (47). PGH was not considered as a direct mediator between inflammatory markers and the infant birthweight z-score, because that would violate an assumption of temporality, given that they were measured contemporaneously in the same blood sample. All regression models were conducted using PROC REG in SAS 9.4 (Cary, NC). We report correlation and regression parameters as statistically significant when attaining an alpha of 0.05.

## Results

### Descriptive statistics

Descriptive statistics for the full sample, and by race, are presented in **Table 1**. Bivariate correlations between pre-pregnancy BMI, PGH, and inflammatory markers for all women are presented in **Table 2**. Black women reported higher pre-pregnancy BMIs than the White women and on average had a lower level of educational attainment. Across all participants, education was significantly associated with adiposity prior to conception (*r* = -0.21, *p* = 0.03). In keeping with one of the primary hypotheses, higher pre-pregnancy BMI was correlated with lower PGH in mid-pregnancy (*r* = -0.22, *p* = 0.02) and higher levels of IL-1Ra (*r* = 0.31, *p* < 0.001), IL-6 (*r* = 0.35, *p* < 0.001), and CRP (*r* = 0.24, *p* = 0.01). The levels of inflammatory proteins were moderately correlated: IL-6 was positively correlated with IL-1Ra (*r* = 0.23, *p* = 0.02) and CRP (*r* = 0.31, *p* = 0.001) and additionally, IL-1Ra was positively correlated with CRP (*r* = 0.24, *p* = 0.01) and IL-8 (*r* = 0.19, *p* = 0.05). Of these three inflammatory proteins, IL-1Ra (*r* = -0.23, *p* = 0.02) and CRP (*r* = -0.20, *p* = 0.04) were inversely correlated with PGH levels whereas IL-8 was positively correlated with PGH (*r* = 0.21, *p* = 0.03).

**Table 1:** Descriptive statistics of the analytic sample.

		Full Sample		Black Women		White Women		*p*-value
		N	Mean (SD)	N	Mean (SD)	N	Mean (SD)	
Pre-pregnancy BMI (self-report)		108	27.4 (6.7)	38	30.2 (7.3)	70	25.9 (5.9)	0.002
Maternal age at the prenatal visit (years)		109	31.5 (5.2)	39	31.5 (6.1)	70	31.5 (4.6)	1.00
Maternal education (years)		109	15.6 (2.7)	39	13.9 (2.2)	70	16.5 (2.5)	<0.001
PGH (natural log)		109	6.5 (0.7)	39	6.6 (0.8)	70	6.4 (0.6)	0.29
IL-1Ra (natural log)		109	5.4 (0.5)	39	5.3 (0.4)	70	5.5 (0.6)	0.02
IL-8 (natural log)		109	0.6 (0.5)	39	0.6 (0.5)	70	0.6 (0.4)	0.37
IL-6 (natural log)		109	6.4 (0.6)	39	6.6 (0.6)	70	6.2 (0.6)	0.003
CRP (natural log)		109	11.8 (11.2)	39	11.5 (10.8)	70	11.9 (11.5)	0.44
Birthweight		106	7.5 (1.1)	37	7.2 (1.2)	69	7.6 (1.0)	0.05
BWz		99	0.0 (0.9)	33	-0.2 (1.0)	66	0.1 (0.9)	0.11
		N	%	N	%	N	%	*p*-value
Infant Sex	Female	54	52.4	22	62.9	32	47.1	0.13
	Male	49	47.6	13	37.1	36	52.9	

**Table 2:** Bivariate correlations between focal predictors among the full sample.

	Pre-pregnancy BMI	Race (Black/ White)	Maternal age	Maternal education	PGH^a^	IL-1Ra^a^	IL-8^a^	IL-6^a^	CRP^a^	Infant’s sex (F/M)	BWz
Pre-pregnancy BMI	--	0.30**	0.11	-0.21*	-0.22*	0.31***	-0.02	0.35***	0.24*	-0.11	0.06
Race (Black/ White)		--	0.00	-0.47***	0.10	-0.22*	0.09	0.28**	-0.07	0.15	-0.16
Maternal age			--	0.31**	-0.03	0.00	0.12	-0.03	0.08	-0.01	-0.10
Maternal education				--	-0.04	0.01	-0.06	-0.14	0.17+	-0.09	-0.15
PGH^a^					--	-0.23*	0.21*	-0.06	-0.20*	0.10	0.06
IL-1Ra^a^						--	0.19*	0.23*	0.24*	-0.13	0.02
IL-8^a^							--	0.15	-0.13	0.01	-0.15
IL-6^a^								--	0.31**	0.05	-0.07
CRP^a^									--	-0.02	-0.04
Infant’s sex (F/M)										--	-0.08
BWz											--

a PGH, IL-1Ra, IL-8, IL-6, and CRP were natural log transformed.

+p<.10 *p<.05 **p<.01 ***p<.001.

Bivariate correlations between focal variables are presented in **Table 3** separately for Black and White women. Examining these estimates, the overall correlation between pre-pregnancy BMI and PGH appeared to be driven by the Black mothers (*r* = -0.44, *p* < 0.01). Similarly, there was a strong association between pre-pregnancy BMI and IL-1Ra among Black women (*r* = 0.68, *p* < 0.001), and to a slightly lesser degree in White women (*r* = 0.32, *p* < 0.01). Finally, preconception BMI was associated with IL-6 (*r* = 0.47, *p* < 0.01) and CRP (*r* = 0.34, *p* = 0.03) only in Black women.

**Table 3:** Bivariate correlations between focal predictors shown separately for Black and White women, above and below the diagonal, respectively.

	Pre-pregnancy BMI	Maternal age	Maternal education	PGH^a^	IL-1Ra^a^	IL-8^a^	IL-6^a^	CRP^a^	Infant’s sex (F/M)	BWz
Pre-pregnancy BMI		0.13	-0.12	-0.44**	0.68***	-0.27	0.47**	0.34*	-0.37*	0.01
Maternal age	0.10		0.40*	-0.09	0.13	0.22	0.12	0.19	-0.16	-0.13
Maternal education	-0.06	0.33**		0.07	-0.17	-0.09	0.19	0.21	-0.17	-0.14
PGH^a^	-0.11	0.02	-0.03		-0.33*	0.38*	-0.18	-0.34*	0.16	0.03
IL-1Ra^a^	0.32**	-0.06	-0.08	-0.17		0.00	0.51***	0.33*	-0.28	0.21
IL-8^a^	0.13	0.04	0.02	0.04	0.33**		0.13	-0.16	-0.13	-0.18
IL-6^a^	0.18	-0.13	-0.11	-0.04	0.25*	0.14		0.28+	-0.22	-0.16
CRP^a^	0.22+	-0.02	0.11	-0.06	0.21+	-0.09	0.40***		-0.07	-0.27
Infant’s sex (F/M)	-0.04	0.09	0.05	0.03	-0.05	0.09	0.11	0.03		0.04
BWz	0.19	-0.08	-0.32**	0.13	-0.10	-0.11	0.04	-0.06	-0.11	

Correlations above the diagonal are within women who self-identified as Black; correlations below the diagonal are within women who self-identified as White.

a PGH, IL-1Ra, IL-8, IL-8, and CRP were natural log transformed.

+p<.10 *p<.05 **p<.01 ***p<.001.

### Main effects among prenatal variables for the full sample

The association between the four inflammatory markers (IL-8, IL-1Ra, IL-6, and CRP) and PGH was examined in a regression model along with other covariates. Only IL-8 evinced a tendency to be directly related to PGH (*β* = 0.20, *p* = 0.05), whereas IL-1Ra (*β* = -0.18, *p* = 0.11), IL-6 (*β* = 0.00, *p* = 0.98), and CRP (*β* = -0.08, *p* = 0.42) were not associated with PGH levels. Therefore, only IL-8 was included in the final model containing the full sample. In this model, self-reported race (*β* = 0544, *p* = 0.03) was positively associated with PGH, whereas pre-pregnancy BMI was negatively associated with PGH levels (*β* = -0.36, *p* = 0.001). IL-8 was positively associated with PGH (*β* = 0.18, *p* = 0.07), although this effect did not reach statistical significance. Together, the included predictors explained 13% of the adjusted variance in PGH, which was statistically significant (*F* (7, 94) = 3.18, *p* = 0.005).

### Stratified analyses

**Figure 1:**
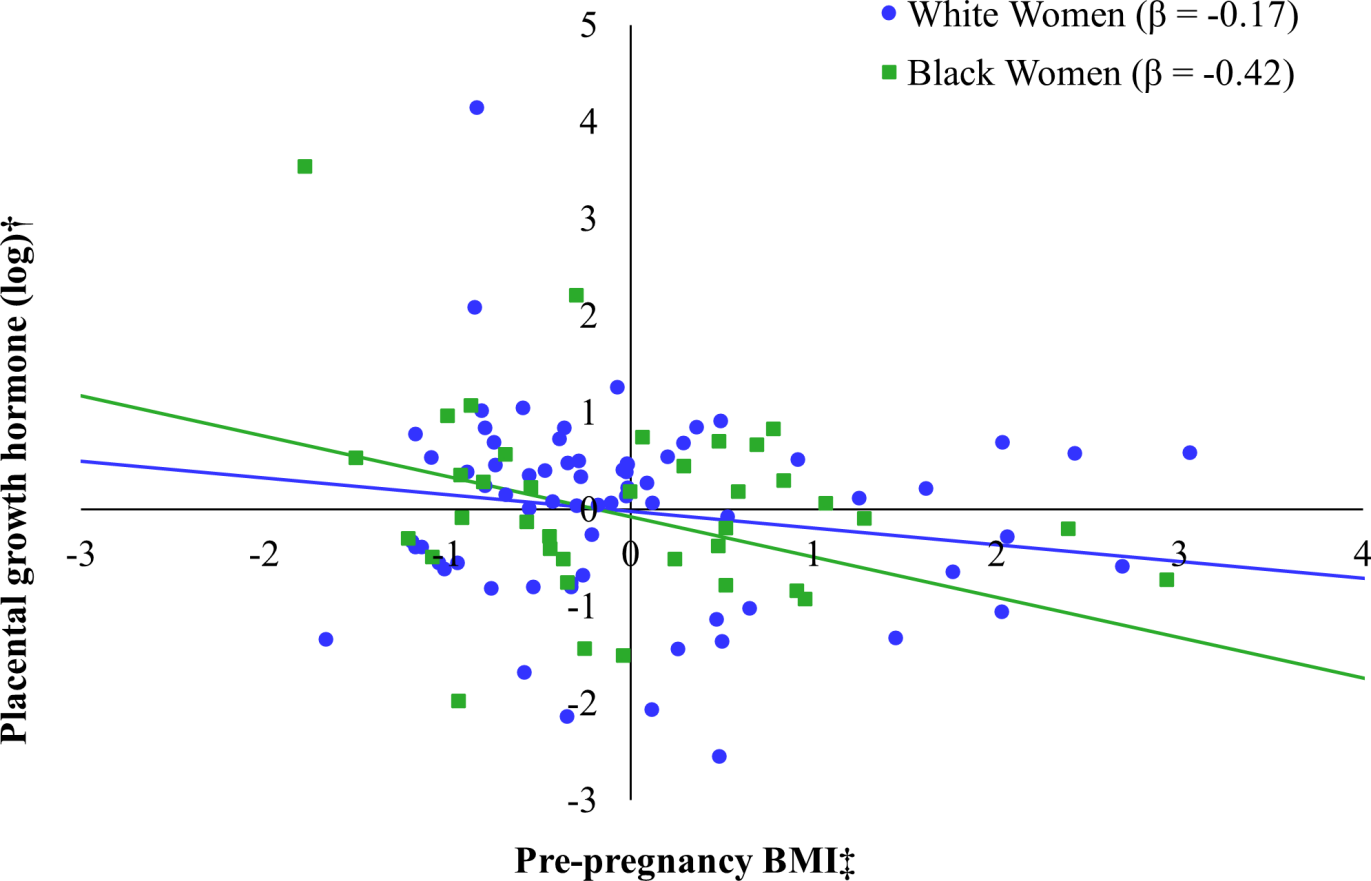
Relation between pre-pregnancy body mass index (BMI) and placental growth hormone (PGH) by maternal race*. *Models additionally adjusted for gestational age at the prenatal visit, maternal age at the prenatal visit, maternal education, IL-8 (log-transformed), and fetal sex. †Placental growth hormone was log transformed. ‡Pre-pregnancy BMI was captured by self-report at the prenatal visit.

**Figure 2:**
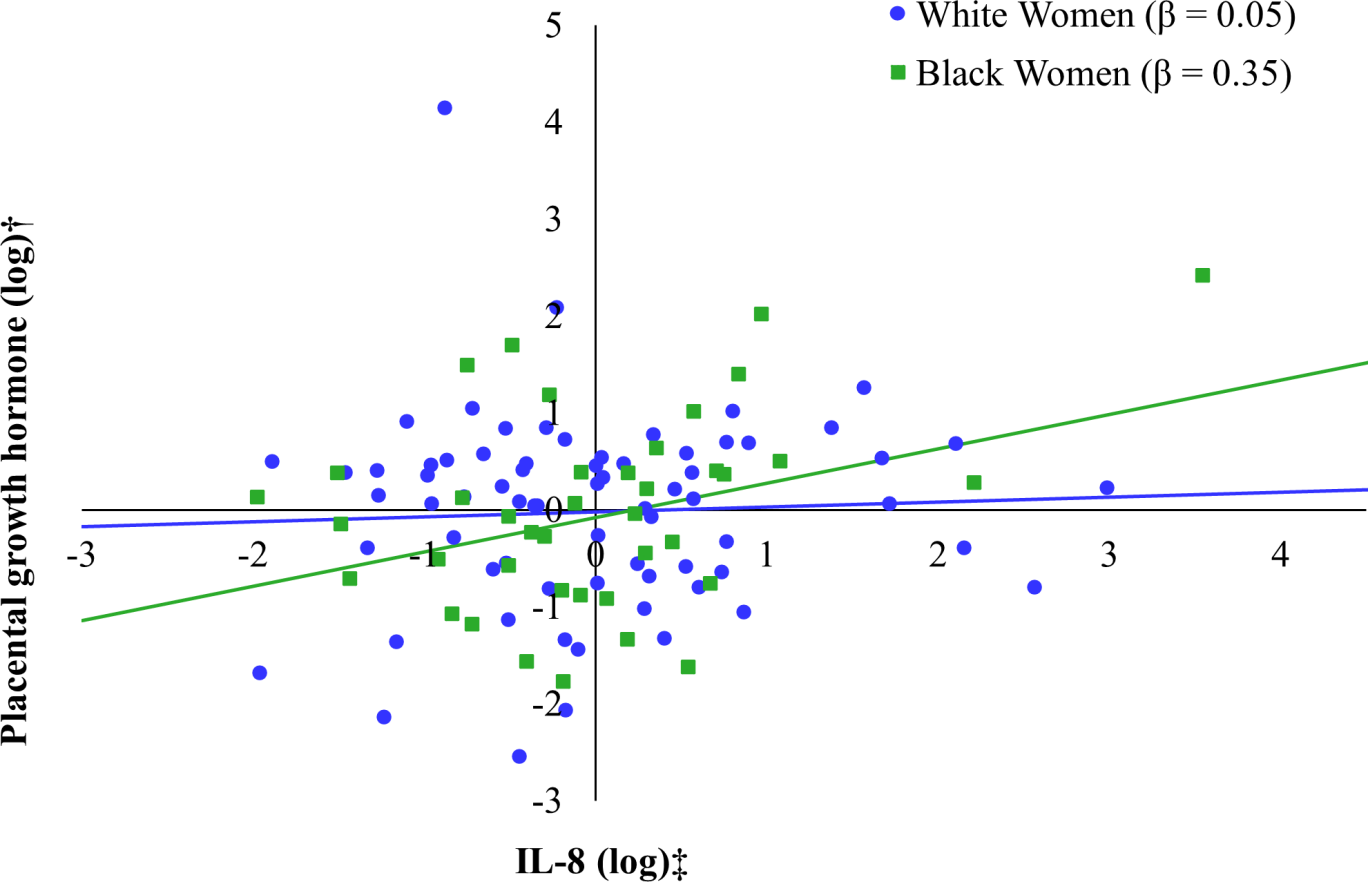
Relation between IL-8 (log-transformed) and placental growth hormone (PGH) by maternal race*. *Models additionally adjusted for gestational age at the prenatal visit, maternal age at the prenatal visit, maternal education, pre-pregnancy BMI (self-report), and fetal sex. †Placental growth hormone and IL-8 were log transformed.

We next examined models stratified by race to address Aim 2 (see **Table 4**). In the regression model stratified to Black mothers, again only IL-8 showed a tendency to be directly related to PGH (*β* = 0.34, *p* = 0.08), whereas IL-1Ra (*β* = 0.03, *p* = 0.89), IL-6 (*β* = -0.02, *p* = 0.94), and CRP (*β* = -0.18, *p* = 0.26) were not associated with PGH levels. Therefore, only IL-8 was included in the final model of Black mothers. IL-8 was positively associated with PGH (*β* = 0.35, *p* = 0.04), whereas pre-pregnancy BMI was negatively associated with PGH (*β* = -0.42, *p* = 0.03). Together, this model explained 39% of the adjusted variance in PGH, which was also statistically significant (*F* (6, 27) = 4.45, *p =* 0.003). In contrast, none of the inflammatory markers were associated with PGH levels when stratifying to only the sample of White mothers. For consistency with the Black sample, we report the regression model including IL-8; however, the combined set of predictors did not account for a significant portion of the adjusted variance in PGH (*F* (6, 61) = 0.42, *p* = 0.87). We illustrate race-related differences in the pattern of associations between BMI and PGH (see **Figure 1**) and between IL-8 and PGH (see **Figure 2**) for Black and White women.

**Table 4:** Multiple linear regressions of PGH^a^ in full sample and stratified by race.

	Full Sample (N=102)	Black Women (N=34)	White Women (N=68)
	β (95% CI)	β (95% CI)	β (95% CI)
Gestational age at prenatal visit	0.16 (-0.04, 0.35)	0.22 (-0.07, 0.51)	0.11 (-0.17, 0.39)
Maternal age	0.00 (-0.20, 0.20)	-0.13 (-0.46, 0.21)	0.04 (-0.24, 0.32)
Maternal education	0.09 (-0.14, 0.32)	0.34 (-0.01, 0.68)+	-0.02 (-0.29, 0.25)
Race (Black vs. White)	0.54 (0.06, 1.02)*	--	--
Pre-pregnancy BMI	-0.36 (-0.57, -0.15)**	-0.42 (-0.78, -0.05)*	-0.17 (-0.44, 0.10)
IL-8^a^	0.18 (-0.02, 0.37)+	0.35 (0.01, 0.70)*	0.05 (-0.21, 0.31)
Infant’s sex (female vs. male)	0.02 (-0.37, 0.40)	0.19 (-0.48, 0.86)	-0.02 (-0.54, 0.50)
*F* (NDF, DDF)	3.18 (7, 94)**	4.45 (6, 27)**	0.42 (6, 61)
Adjusted R^2^	0.13	0.39	0.00

a PGH, IL-1Ra, IL-8, IL-6, and CRP were log-transformed.

+p<.10 *p<.05 **p<.01 ***p<.001.

### Main effects of PGH, BMI, and inflammatory measures on birthweight Z-score

The next models evaluated associations between the same set of predictors and fetal growth as indexed by the infant’s birthweight z-score (BWz), which corrected for the sex of the infant and gestational age at term (see **Table 5**). When estimating the model with the full sample, mid-pregnancy PGH was positively associated with BWz, although not statistically significant (PGH: *β* = 0.17, *p* = 0.06), whereas maternal IL-8 was negatively associated with the infant’s BWz (*β* = -0.19, *p* = 0.03). There was also an influence of race and maternal education, reflecting the lower birthweight of Black infants and the association of a smaller BWz among babies born to women with a lower educational attainment (Black vs. White race: *β* = -0.68, *p* < 0.01; education: *β* = -0.29, *p* < 0.01). This model explained 11% of the adjusted variance in BWz (*F*(6, 91) = 3.04, *p* = 0.009).

**Table 5:** Multiple linear regressions of BWz in full sample and stratified by race.

	Full Sample (N=98)	Black Women (N=32)	White Women (N=66)
	β (95% CI)	β (95% CI)	β (95% CI)
Maternal age	-0.02 (-0.20, 0.17)	0.07 (-0.35, 0.49)	-0.02 (-0.24, 0.20)
Maternal education	-0.29 (-0.49, -0.08)**	-0.32 (-0.74, 0.10)	-0.27 (-0.49, -0.05) *
Race (Black vs. non-Black)	-0.68 (-1.13, -0.23)**	--	--
Pre-pregnancy BMI	0.16 (-0.04, 0.36)	0.01 (-0.41, 0.43)	0.21 (-0.01, 0.43)+
IL-8^a^	-0.19 (-0.36, -0.01)*	-0.40 (-0.82, 0.02)+	-0.12 (-0.33, 0.08)
PGH^a^	0.18 (-0.01, 0.36)+	0.26 (-0.19, 0.70)	0.13 (-0.08, 0.34)
F (NDF, DDF)	3.04 (6, 91)**	1.06 (5, 26)	2.55 (5, 60)*
Adjusted R^2^	0.11	0.01	0.11

a IL-1Ra, IL-8, IL-6, CRP, and PGH were natural-log transformed.

+p<.10 *p<.05 **p<.01 ***p<.001.

**Figure 3:**
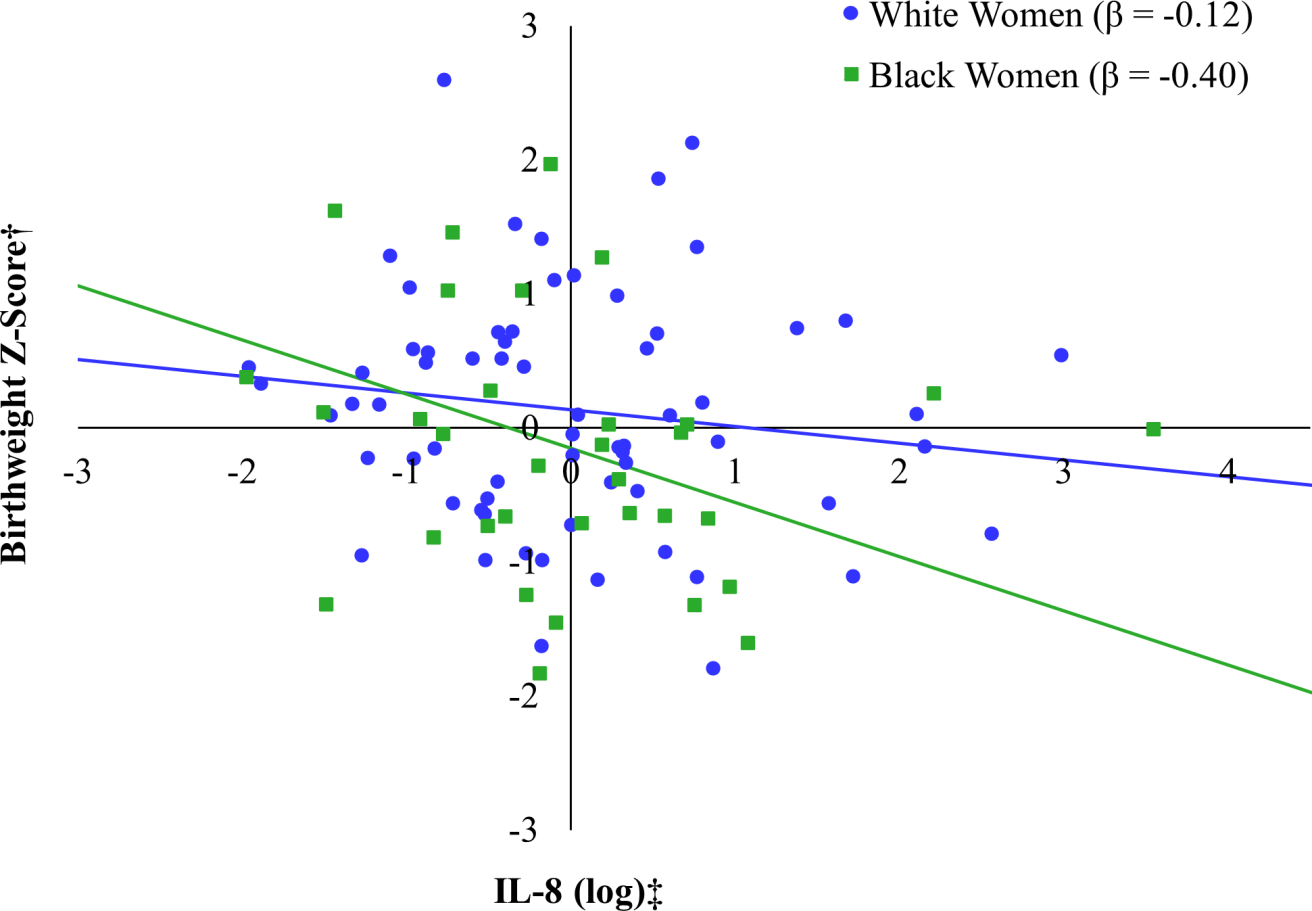
Relation between IL-8 (log) and birthweight Z-score by race*. *Models additionally adjusted for maternal age at the prenatal visit, maternal education, pre-pregnancy BMI (self-report), and PGH (log-transformed). †Birthweight Z-score accounts for gestational age at birth and fetal sex. ‡IL-8 was log transformed.

**Figure 4:**
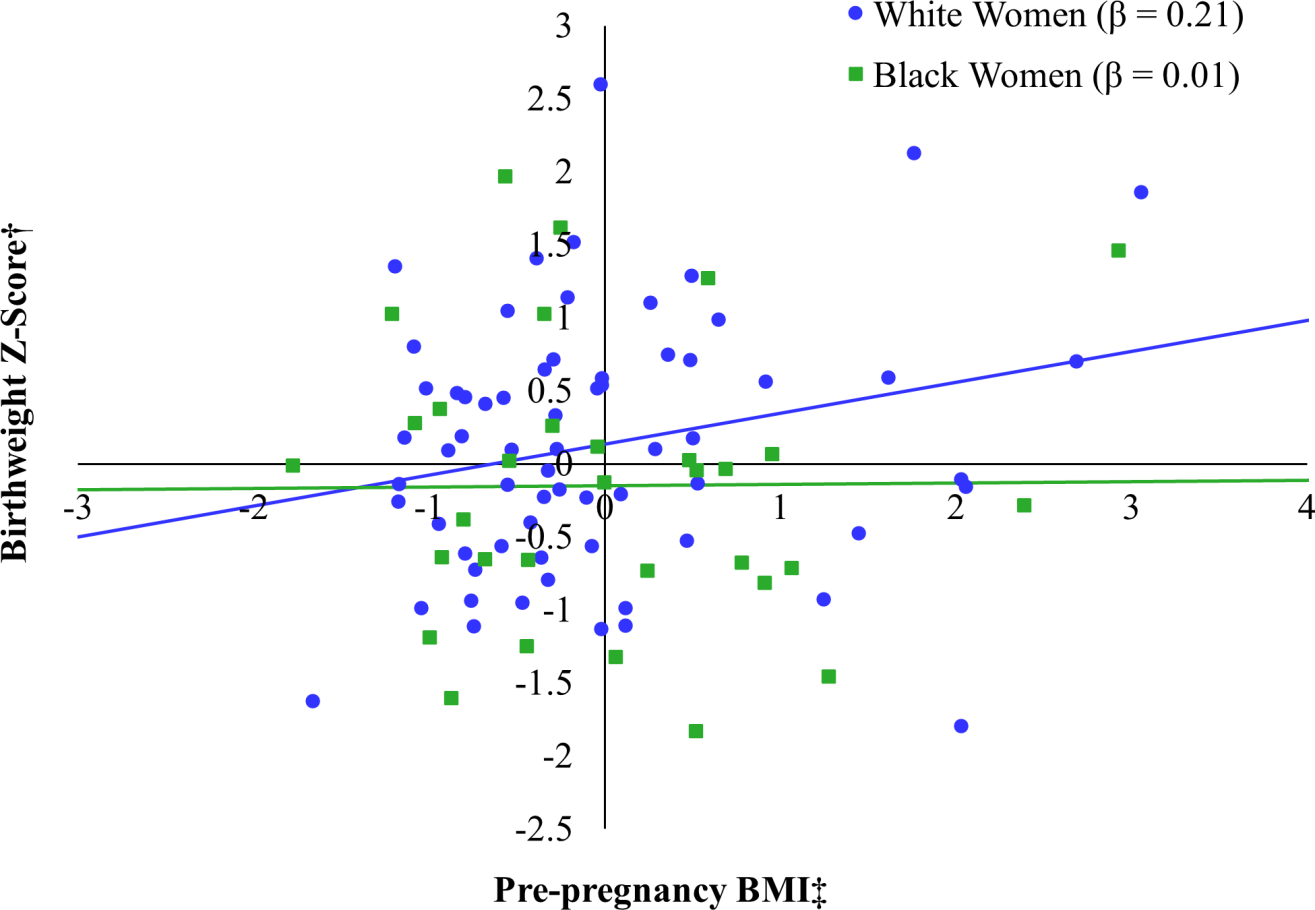
Relation between pre-pregnancy BMI and birthweight Z-score by race*. *Models additionally adjusted for maternal age at the prenatal visit, maternal education, IL-8 (log-transformed), and PGH (log-transformed). †Birthweight Z-score accounts for gestational age at birth and fetal sex. ‡Pre-pregnancy BMI was captured by self-report at the prenatal visit.

When stratified to the Black participants only, IL-8 was negatively associated with BWz, although this trend approached but did not attain statistical significance (*β* = -0.40, *p* = 0.06) (see **Figure 3**). In fact, none of the predictors significantly predicted infant BWz when considered individually (*F*(5, 26) = 1.06, *p* = 0.41). When stratifying to White participants only, maternal educational attainment was negatively associated with infant BWz (*β* = -0.26, *p* = 0.02) and BMI was positively associated with infant BWz (see **Figure 4**), although this trend did not attain the cutoff for statistical significance (*β* = 0.21, *p* = 0.06). This model explained 11% of the adjusted variance in infant BWz, which was statistically significant (*F*(5, 60) = 2.55, *p* = 0.04).

### Testing indirect effects of maternal BMI on infant BWz through PGH

Finally, we tested whether there might be indirect effects of maternal adiposity on BWz through maternal PGH. We estimated total and indirect effects in the full sample and among participants stratified by race (see **Table 6**). Pre-pregnancy BMI predicted PGH (*β* = -0.33, *p* < 0.02) and PGH was associated with BWz, although not statistically significant (*β* = 0.18, *p* = 0.06) in the full sample. However, the indirect effect of BMI on BWz *via* PGH only approached significance (*β* = -0.06, *p* = 0.11). There wasn’t evidence for indirect effects of BMI on BWz through PGH in either of the subgroups stratified by race.

**Table 6:** Testing cascading effects of maternal pre-pregnancy BMI on infant BWz through PGH^a^.

Outcome is birthweight z-score*	Full Sample (N=98)	Black Women (N=32)	White Women (N=66)
	β (95% CI)	β (95% CI)	β (95% CI)
Pre-pregnancy BMI→ PGH	-0.33 (-0.54, -0.12)**	-0.41 (-0.75, -0.08)*	-0.14 (-0.40, 0.12)
Pre-pregnancy BMI→ BWz	0.16 (-0.04, 0.36)	0.01 (-0.41, 0.43)	0.21 (-0.01, 0.43)+
PGH → BWz	0.18 (-0.01, 0.36)+	0.26 (-0.19, 0.70)	0.13 (-0.08 0.34)
BMI → PGH → BWz (indirect)^b^	-0.06 (-0.12, -0.01)+	-0.11 (-0.31, 0.05)	-0.02 (-0.06, 0.02)
Pre-pregnancy BMI→ BWz (total) ^b^	0.10 (-0.09, 0.29)	-0.10 (-0.48, 0.28)	0.19 (-0.02, 0.41)+
Total effect model *F* (NDF, DDF)	2.86 (5, 92)*	0.96 (4, 27)	2.78 (4, 61)*
Total effect model R^2^	0.13	0.12	0.15

a All mediation models adjusted for maternal age at the prenatal visit, maternal education, and log-transformed IL-8. The mediation model with the full sample additionally adjusted for binary race.

b For indirect and total effects, 95% CIs refer to the bootstrapped lower and upper confidence intervals for the effects.

+p<.10 *p <.05 **p <.01 ***p <.001.

### Sensitivity analysis: Main effects among prenatal predictors

Finally, to examine whether any outlier values influenced the observed associations, BMI, PGH, inflammatory markers values that were more than 3 standard deviations above the mean (for PGH and inflammatory markers—the log-transformed mean) were excluded, and the models re-estimated. In total, seven outlier values were set to missing, though no mother was an outlier value on more than one measure. Mothers who had outlier values were not different from mothers without outlier values with respect to self-reported race, report of high blood pressure in pregnancy, age, education, or birthweight. When modeling PGH for the full sample and including binary race as a predictor, pre-pregnancy BMI continued to be negatively associated with PGH (*β* = -0.25, *p* = 0.02) and mothers who were Black had higher PGH than White mothers (*β* = 0.58, *p* = 0.02). Among the Black participants, BMI also continued to be negatively associated with PGH (*β* = -0.36, *p* = 0.06), albeit now not reaching statistical significance. In addition, the association between IL-8 and PGH became non-significant after removing the highest values (*β* = 0.23, *p* = 0.21). This model explained 22% of the adjusted variance in PGH. However, taking the small number of biomarker outlier values into consideration, there were no substantive changes in the models estimating BWz.

## Discussion

Our findings have affirmed many previous studies reporting high levels of PGH in maternal circulation during the second and third trimesters (45, 48, 49). In keeping with our initial hypothesis, the levels of PGH were associated with preconception adiposity, although this effect was moderated by race, with a prominent effect evident only among the Black women. Similarly, the association between IL-8 levels and PGH in the pregnant women was more pronounced among participants who self-identified as Black. After considering a number of covariates, including the gestational timing when blood was collected and maternal educational attainment, the joint set of predictors which included adiposity and IL-8 accounted for a large proportion of the variance of PGH in Black mothers (39%), whereas the overall regression models were nonsignificant and did not account for the variation among in the White women.

The placental synthesis and release of PGH has been the focus of extensive clinical and basic science research for over three decades (4, 8, 50). It is known that PGH levels rise progressively across pregnancy, supplanting the release of GH by the maternal pituitary, and act on the mother’s lipid metabolism and glucoregulation. While PGH has been detected in amniotic fluid and fetal circulation (51–53), it is believed that the primary influence on fetal growth is indirect *via* stimulating nutrient availability and placental transfer of resources to the fetus (54). PGH levels have been more reliably linked to fetal growth rates when size is measured contemporaneously *via* ultrasound assessments, and the strength of this association is often found to lessen by the time of delivery because multiple factors influence the rapid growth of the fetus during the final weeks of pregnancy, including the length of pregnancy and gestational age at delivery. In keeping with this perspective, we did not find a significant correlation between PGH levels measured in mid-pregnancy and infant birthweight later at delivery. However, our finding of lower PGH in women who reported having larger BMIs prior to conception would be congruent with reports of lower PGH in women who are more likely to progress to gestational diabetes and hypertension/eclampsia (54, 55). The fact that we did not find an association between PGH levels and preterm birth or small-for-gestational-age infant is likely to be more of a reflection of our study’s exclusion criteria, which selected for women with generally healthy pregnancies and gestation lengths of normal duration. Similarly, our inclusion/exclusion criteria would have also lessened the chance of finding a race-related difference in the prevalence of premature births and/or infants of low birthweight. In larger population-level analyses, more adverse pregnancy outcomes and health disparities are typically found among Black women (56).

In addition to determining that the levels of PGH are high in maternal circulation, we also replicated previous reports of an increase in the levels of several proinflammatory proteins in the later stages of pregnancy (3). In particular, we found high levels of IL-1Ra (57). Prior research has indicated that a portion of this IL-1Ra is likely synthesized and released by the placenta, and it is believed to act as a circulating buffer to moderate the inflammatory actions of its ligand, IL-1, and the synergistic effects of other proinflammatory cytokines that might be stimulated by IL-1, such as IL-6 and TNFalpha (58). For both Black and White participants, maternal adiposity prior to conception was positively correlated with the level of IL-1Ra at mid-pregnancy. This suggests a general influence of obesity and gestational weight gains on proinflammatory activity because the release of soluble IL-1Ra into circulation is usually associated with an increase in the recent activity of proinflammatory cytokines. In addition, across women of both racial groups, IL-1Ra was positively correlated with IL-6 (0.51 and 0.25, in Black and White women, respectively).

IL-8 was also selected for this analysis as a bioindicator of placentokine activity (59) because it is known to be constitutively expressed by trophoblasts and synthesized and secreted by the fully developed placenta (17, 60). We replicated reports of high levels of IL-8 in maternal circulation during mid-pregnancy (61). Others have found that IL-8 will increase further until term and it can reach extremely high levels during labor (62). The progressive rise across pregnancy has led some to suggest that IL-8 serves as a contributory signal which helps to stimulate labor (63), but it is likely to have other functions given that the chemotactic actions of IL-8 in the nonpregnant individual are important in neutrophil recruitment and activation in response to traumatic injury and damage to epithelial tissue (64). Although the levels of IL-8 were not different between Black and White women, the pattern of associations with the other measures revealed a number of specific differences. For example, there was a significant correlation between IL-8 and PGH levels among Black women, which was not evident among White women. This difference was in keeping with the fact that preconception adiposity in the Black women was more strongly associated with both lower PGH and lower IL-8 levels during pregnancy. Conversely, among the White women, IL-8 was positively associated with IL-1Ra levels. These race-related differences may reflect either variation in the contribution of different tissue sources for these soluble proteins released into circulation or perhaps a difference in regulatory feedback, including the immunomodulatory actions of the soluble receptor antagonists in maternal circulation.

In addition to examining the influence of sociodemographic factors and preconception adiposity on maternal physiology during pregnancy, we were also interested in whether PGH or the other cytokine biomarkers might be associated with fetal growth and birthweight at term. Birthweight was examined and a z-scored adjustment was employed to take into account the infant’s gestational age at delivery and the tendency for a female/male difference in size at term. There was a significant racial difference in absolute birthweight, with lower weights in Black infants as compared to White infants. The size of the babies born to White mothers also revealed a stronger influence of maternal educational attainment, which was not as evident among the Black women. In the current analyses, we were not able to replicate previous reports of an association between low levels of PGH during pregnancy and small-for-gestational age infants. However, the absence of a stronger effect of PGH likely reflects our study exclusion criteria, which prevented the inclusion of premature births (i.e., less than 36 weeks, 4 days), where a stronger association between low PGH and low birthweight has typically been found. At least one study has reported that PGH levels may be associated with fetal size when assessed contemporaneously by ultrasound measurement, but that relation likely becomes obscured by rapid fetal growth at the end of gestation and is not as readily apparent when assessed by birthweight at term (45). We did not observe significant differences in the levels of maternal PGH or cytokines between women gestating a female or male baby but given that two studies have found that placental cytokine levels are affected by the sex of the fetus, it could be another contributing factor (61, 65).

### Limitations

Although this analysis has generated a number of new findings, we should also acknowledge a several limitations. In particular, the physiological assessments were based on a single blood sample at one point in pregnancy, and thus it was not possible to examine temporal changes over time, which might have been more revealing of relationships with maternal adiposity. In our statistical analyses, we corrected for the gestational age when the blood was collected during mid-pregnancy, but it should be acknowledged that the data collection was spread over many weeks in mid-pregnancy when there are likely to be dynamic changes in some of these biomarkers. PGH was selected because it is synthesized by the placenta and known to influence lipid metabolism and glucoregulation, but the physiology of pregnancy and energy metabolism is complex (66). Thus, many other key factors were not included in our analyses, including adipokines like leptin, which are known to be associated with obesity and with cytokine biology (67–69). In addition, our measure of adiposity may not have been sufficiently fine-grained because the women’s weight and height prior to pregnancy was obtained *via* self-report measure, rather than medical records. Additionally, BMI may not be the best measure to reflect body fat as it does not account for racial and ethnic differences in body phenotype, including a higher BMI due to muscularity (70). It is also known that gestational weight gain across pregnancy may be an equally important factor because it often accentuates the adverse effects of maternal obesity on pregnancy and fetal outcomes (71). Due to in-person data collection restrictions as a result of COVID-19, our analytic sample was restricted to participants whose prenatal visit was conducted prior to March 2020 (as the blood sample was drawn in the laboratory), and the study sample is therefore limited in its size. In particular, the statistical models used to evaluate race-related differences were constrained by the relatively small number of participants in the stratified samples, limiting the power to detect significant effects should they exist. The sample size along with our exclusion strategy, which selected for longer-term pregnancies, also likely contributed to the absence of premature births and small-for-gestational age infants, where the effects of abnormal placental physiology and high levels of inflammatory activity may be more prominent (45).

### Conclusion and future directions

Notwithstanding these limitations, this analysis is foundational for future research investigating the influence of sociodemographic factors on maternal health and placenta-associated biomarkers that can affect maternal and infant wellbeing during pregnancy and beyond (71). We have now linked PGH with preconception adiposity, providing a new pathway to consider in research investigating the adverse effects of maternal obesity on pregnancy outcomes. Furthermore, our analysis suggests that in addition to the effects of maternal adiposity on placental structure (3), there may be an influence on the synthesis and release of soluble mediators like PGH, with the potential for transplacental effects on fetal growth and metabolism. This study also highlighted the importance of considering the possibility of race-related differences in the pattern of associations between health-related physiological indices. The influence of socio-demographic factors and diet on obesity among women of child-bearing age is critical to consider in research on maternal and child health (56, 72).

## Data availability statement

The original contributions presented in the study are included in the article/Supplementary Material. Further inquiries can be directed to the corresponding author.

## Ethics statement

The studies involving human participants were reviewed and approved by Institutional Review Board; University of North Carolina at Chapel Hill. Written informed consent to participate in this study was provided by the participants’ legal guardian/next of kin.

## Author contributions

CW: Conceptualization, writing – original draft, review & editing. AW: Formal analysis, writing – original draft, review & editing. VG: References, writing – review & editing. CC: Conceptualization, Writing – Original Draft, Review & Editing. SS: Conceptualization, resources, supervision, project administration, funding acquisition; writing – original draft, review & editing. All authors contributed to the article and approved the submitted version.
